# Combined serum biomarker analysis shows no benefit in the diagnosis of periprosthetic joint infection

**DOI:** 10.1007/s00264-020-04731-6

**Published:** 2020-07-25

**Authors:** S. M. Klim, F. Amerstorfer, G. Glehr, G. Hauer, M. A. Smolle, L. Leitner, A. Leithner, M. Glehr

**Affiliations:** 1 Department of Orthopaedics and Trauma, Medical University of Graz, Auenbruggerplatz 5, 8036, Graz, Austria; 2 Statistical Bioinformatics Department, University of Regensburg, Am BioPark 9, 93053 Regensburg, Germany

**Keywords:** Biomarker, Periprosthetic joint infection, Diagnostics, Revision joint arthroplasty

## Abstract

**Purpose:**

In many cases, the diagnosis of a periprosthetic joint infection (PJI) consisting of the clinical appearance, laboratory tests, and other diagnostic tools remains a difficult task. Single serum biomarkers are easy to collect, are suitable for periodical assessment, and are a crucial tool in PJI diagnosis, but limited sensitivity or specificity is reported in literature. The aim of this study was to combine the best-performing single serum biomarkers into a multi-biomarker model aiming to improve the diagnostic properties.

**Methods:**

Within a 27-month period, 124 surgical procedures (aseptic or septic revision total knee arthroplasty (TKA) or total hip arthroplasty (THA)) were prospectively included. The serum leukocyte count, C-reactive protein (CRP), interleukin-6, procalcitonin, interferon alpha, and fibrinogen were assessed 1 day prior to surgery. Logistic regression with lasso-regularization was used for the biomarkers and all their ratios. After randomly splitting the data into a training (75%) and a test set (25%), the multi-biomarker model was calculated and validated in a cross-validation approach.

**Results:**

CRP (AUC 0.91, specificity 0.67, sensitivity 0.90, *p* value 0.03) and fibrinogen (AUC 0.93, specificity 0.73, sensitivity 0.94, *p* value 0.02) had the best single-biomarker performances. The multi-biomarker model including fibrinogen, CRP, the ratio of fibrinogen to CRP, and the ratio of serum thrombocytes to CRP showed a similar performance (AUC 0.95, specificity 0.91, sensitivity 0.72, *p* value 0.01).

**Conclusion:**

In this study, multiple biomarkers were tested for their diagnostic performance, with CRP and fibrinogen showing the best results regarding the AUC, accuracy, sensitivity, and specificity. It was not possible to further increase the diagnostic accuracy by combining multiple biomarkers using sophisticated statistical methods.

## Introduction

In many cases, periprosthetic joint infection (PJI), a complication responsible for 14.8% of failed total knee arthroplasties (TKA) and 7.5% of total hip arthroplasties (THA), can be a simple diagnosis [1]. The reason for ongoing research into the diagnosis of PJI is complex cases with often unspecific symptoms. With the rising number of primary TKA and THA, these cases will become more frequent [2]. A reflection of these diagnostic difficulties is the various PJI definitions, with the Musculoskeletal Infection Society (MSIS), the Infectious Disease Society of North America (IDSA), and the modified Zimmerli criteria (EBJIS) being the most frequently used [3–5].

Besides the clinical appearance and physical examination of a joint, PJI biomarkers are crucial for every diagnostic protocol. These biomarkers are usually divided into serum and synovial biomarkers with considerable advantages and disadvantages in each group. A major benefit of serum biomarkers, when compared with synovial fluid biomarkers, is the availability. Blood, in contrast to synovial fluid, can be collected safely with no additional risk of iatrogenic infection of the joint. In combination with generally low costs, this allows periodical assessment and diagnostics as well as treatment monitoring. Many serum biomarkers with different properties have been described in the literature, with leukocyte count, C-reactive protein (CRP), interleukin-6 (IL-6), procalcitonin (PCT), and interferon alpha (IF-alpha) being some of the most common ones [6–8].

However, serum biomarkers have some disadvantages that must be considered. Most serum biomarkers have shown either a high sensitivity or specificity in the literature [6, 7, 9]. Another problem which affects serum markers more than synovial markers is the interference bias when a patient is suffering from another disease that leads to elevated inflammatory markers. Additionally, a recent systematic review reported an inferior diagnostic performance of serum biomarkers in chronic PJI cases when compared with synovial biomarkers [10].

Therefore, the study aim was to calculate a model with multiple serum biomarkers in order to combine their individual strengths in sensitivity and specificity. The purpose was to answer the question, if an optimal combination of serum biomarkers would significantly improve the diagnostic performance and surpass the single best serum test for a PJI.

## Methods

Eighty-eight participants with 130 surgical procedures (aseptic or septic revision TKA or THA) were prospectively included within a 27-month period at a single institution. The serum leukocyte count, CRP, IL-6, PCT, IF-alpha, and fibrinogen were investigated. Patients with chronic inflammatory diseases or disorders of the immune system, obesity (BMI > 30), viral infections, malignancies, heavy smoking (25 or more cigarettes per day), and renal (CKD IV or worse) or hepatic failure were excluded. Prior to the final analysis, four patients with six procedures had to be excluded due to an incomplete data set. The study protocol was approved by the institutional review board and written informed consent was collected from all participants. The methods were carried out in accordance with relevant guidelines and regulations.

To determine the target serum biomarkers, blood was taken one day prior to surgery and analyzed according to our institutional standard: CRP, immune turbidimetry of lithium-heparin blood (normal: 5.0 mg/L); leukocyte count, flow cytometry with EDTA plasma (normal range, 4.4–11.3 G/L); fibrinogen, coagulometry of sodium citrate blood (normal range 210–400 mg/dl); procalcitonin and IL-6, commercially available kits (Elecsys BRAHMS PCT and IL-6 Kit; Roche Diagnostics, Mannheim, Germany); and IFN-a, commercially available ELISA assay (detection limit: 1 pg/mL, normal < 260 pg/mL; Bender MedSystems GmbH, Vienna, Austria).

Five tissue samples were collected intraoperatively from different locations with macroscopic signs of infection around the joint. Each of the five tissue samples is split into five microbiologic and histologic samples and numbered according to a standardized protocol to receive corresponding histologic and microbiologic results. Microbiologic samples were put in a brain-heart infusion and sent for aerobic and anaerobic cultivation and held for a minimum of ten days.

After the data collection, a blinded researcher divided the cases into two groups: group A with confirmed PJI and group B without PJI. This was done based on the MSIS criteria for PJI, with fistulation of the prosthesis or a pathogen isolated by culture from at least two separate samples being major criteria (one positive). The minor criteria (three out of six) for the presence of a PJI were elevated CRP, elevated synovial leukocyte count, presence of purulence in the affected joint, elevated synovial neutrophil percentage, isolated microorganism in one culture, or more than five neutrophils per high-power field in five high-power fields [3].

### Statistical analysis

In order to calculate an optimal multi-biomarker model which would later be tested on a new set of data, the samples were randomly split into a training (75%) and a test (25%) set. Univariate logistic regression was performed, and by using the Youden index [11] derived from the ROC curve, the optimal cutoff values were found. The cutoff values were then applied to the test set and performances assessed. The AUC from the ROC curve and the *p* value of the logistic regression on the training and test set were recorded.

In order to find a possible combination of markers, all missing observations were first imputed by the mean of the existent values. All possible ratios of the features PCT, CRP, leukocytes, IL-6, IF-alpha, fibrinogen, and serum thrombocytes were then calculated and included as new features. Finally, a logistic regression with lasso-regularization was used [12] on all those features in a cross-validation approach on the training samples only. Cross-validation repeats the holdout method multiple times to receive more valid performance estimates. The cutoff was again estimated on the cross-validated training probabilities using the Youden index. On the final model using all samples of the training set, a cutoff was first determined, whereupon the model and the cutoff were applied to the validation set. Again, the AUC and *p* values of those predictions are reported. All statistical analyses were performed using the free software R, Version 3.6.1 (R Core Team, R Foundation for Statistical Computing, Vienna, Austria).

## Results

**Fig. 1:**
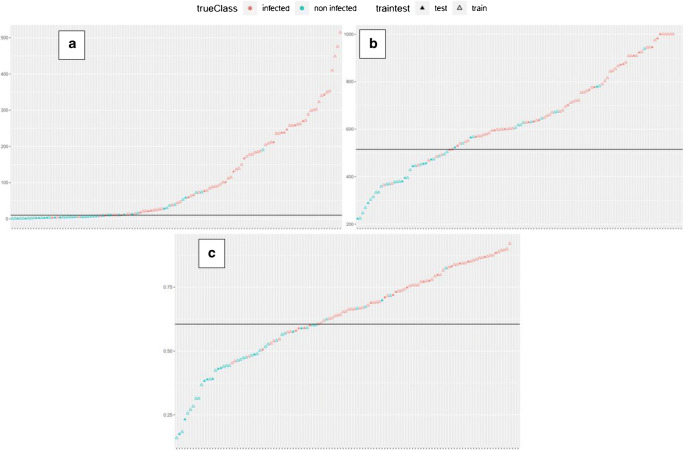
The performances in PJI diagnosis of (**a**) C-reactive protein (CRP), (**b**) fibrinogen, and (**c**) the multi-biomarker analysis for each case on the training (empty triangles, 75% of samples) and test set (full triangles, 25% of samples). PJI-positive cases (red) and PJI-negative cases (blue). The black line displays the respective cutoff values (CRP 10.3 mg/L; fibrinogen 515 mg/dl; multi-biomarker 0.63)

**Fig. 2:**
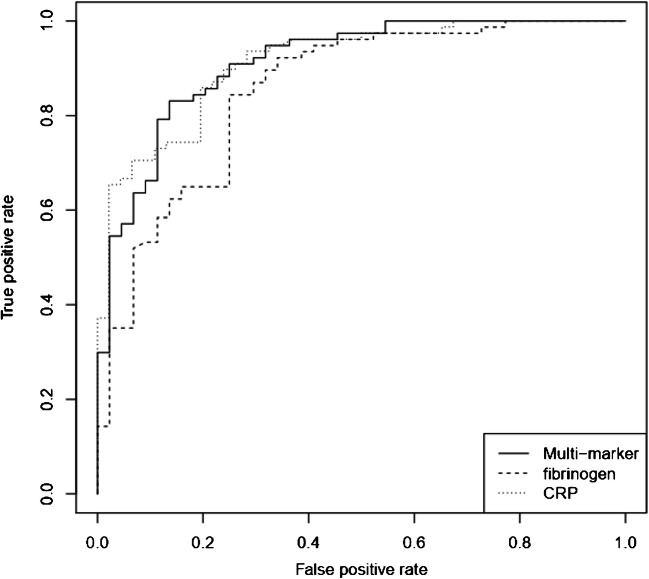
Receiver operating curve (ROC) of the multi-biomarker model compared the two best-performing single biomarkers in PJI diagnosis (C-reactive protein (CRP), fibrinogen). The area under the curve (AUC) has shown no significant differences regarding the diagnostic performance: multi-biomarker model 0.95, CRP 0.91, fibrinogen 0.93

In total, 124 procedures (84 patients) were included in the final analysis (Table 1). The univariate analysis results for the tested serum biomarkers and the performance of the multi-biomarker model including the cutoff were as depicted in Table 2. CRP, and fibrinogens were the best-performing biomarkers regarding the diagnosis of a PJI (*p* < 0.05). While IL-6 was also a statistically significant marker (*p* = 0.03), leukocytes (*p* = 0.07), PCT (*p* = 0.13), and IF-alpha (*p* = 0.99) showed no significant diagnostic value. The performances of the training and test sets (75 and 25%, respectively) of the two best single biomarkers, CRP and fibrinogen, as well as the multi-biomarker model are visible in Fig. 1. The ROC curves of CRP, fibrinogen, and the multi-biomarker model are shown in Fig. 2. The final cross-validated model (respective weights in brackets) is a weighted signature which uses an intercept (−0.24370), the ratio of fibrinogen to CRP (−0.00002), serum thrombocytes to CRP (−0.00632), CRP (0.00242), and fibrinogen (0.00121). More CRP or more fibrinogens increase infection probability. The higher the ratio between fibrinogen and CRP, the less likely is an infection. The higher the ratio between serum thrombocytes and CRP, the less likely is an infection. To get the probability, we calculated the logistic function of the signatures weighted sum. There was no significant improvement when comparing the multi-biomarker model to the best single-biomarker performance.

**Table 1:** Background characteristics of patients included in the final analysis

	Total	PJI positive	PJI negative
Number of patients/procedures	84/124	55/78	29/46
Gender male:female (female %)	38:46 (55%)	26:29 (53%)	12:17 (59%)
Mean age (years)	65.5 ± 15.3	65.7 ± 15.8	65.1 ± 14.6
Affected joint			
Knee (%)	68 (54.8%)	47 (60%)	21 (44.8%)
Hip (%)	56 (45.2%)	31 (40%)	25 (55.2%)
Mean duration of surgery (minutes)	107 ± 59	101 ± 58	119 ± 61

**Table 2:** Univariate analysis results for PJI diagnosis of the tested serum biomarkers and the multi-biomarker model

	AUC	Accuracy	Specificity	Sensitivity	Cutoff	*p* value
CRP	0.91	0.81	0.67	0.90	10.3 mg/L	0.0034
Fibrinogen	0.93	0.86	0.73	0.94	515 mg/dl	0.0196
Leukocytes	0.86	0.61	0.92	0.42	8.17 G/L	0.07
Interleukin-6	0.8	0.74	0.7	0.77	5.7 pg/ml	0.03
Interferon alpha	0.36	0.39	0.82	0	39 pg/ml	0.99
Procalcitonin	0.81	0.6	0.9	0.4	0.1 ng/ml	0.13
Multi-biomarker	**0.95**	**0.79**	**0.91**	**0.72**	**0.63**	**0.01**

### PJI-positive group

*Staphylococci* were responsible for PJI in 30 cases (55% of detected bacteria) while in 16 of 55 cases (29%), no bacteria could be isolated. In the PJI group, antibiotics were given for six weeks post-operative. A two-stage revision (i.e., removal of the prosthesis and temporary non-articulating polymethyl methacrylate (PMMA) spacer implantation) was performed in 33 cases. This was counted as one procedure, with serum markers and other samples being taken at the first operation. Debridement, antibiotics, and implant retention (DAIR) was performed in 32 cases. The thirteen remaining cases were spacer exchanges (five cases), Girdlestone resections (three cases), a reimplantation of an endoprosthesis (three cases) as well as one stem exchange and one inlay exchange.

### PJI-negative group

*Staphylococci* species were isolated in three, *Enterobacter cloacae* in one, and *Proteus mirabilis* in one of those 29 patients from the postoperative Redon drainage. Since there were no other infection criteria and the patients remained PJI free, the findings were interpreted as a contamination. Surgical procedures in the aseptic group were a spacer explantation and the reimplantation of an endoprosthesis in 19 cases, a spacer implantation after the removal of the endoprosthesis in 16 cases, four mobile part exchanges, three arthrodesis of the knee, two spacer exchanges, and one Girdlestone plastic as well as one endoprosthesis explantation.

## Discussion

In the diagnosis of a PJI, serum biomarkers are a valuable tool for the attending physician as they can be easily obtained and periodically assessed. In this prospective study, the serum leukocyte count, CRP, IL-6, PCT, IF-alpha, and fibrinogen were investigated for their diagnostic performances. It was determined that the elevated levels of CRP, fibrinogen, and IL-6 significantly correlate with the presence of a PJI (*p* < 0.05). In contrast, serum leukocyte count, PCT, and IF-alpha had limited diagnostic value in this study cohort. The mathematical combination of the best-performing biomarkers included CRP, fibrinogen, and their ratio as well as the ratio of thrombocytes to CRP in the final model. However, this could not improve the diagnostic accuracy.

While CRP is an established PJI biomarker, fibrinogen, a glycoprotein usually known for its important role in the coagulation cascade, also mediates the inflammation process and has shown a surprisingly good specificity and sensitivity for PJI in this study [13, 14]. Comparable results have been reported in the recent literature [15–17]. In addition, D-dimer, another product of the coagulation cascade, has also shown promising results in the recent literature as a PJI biomarker [18, 19]. It remains to be seen whether these markers will make an impact in routine PJI diagnostic protocols. As reported in various publications, PCT had a high specificity with a rather low sensitivity for PJI in our cohort. Therefore, PCT remains a good rule-in test yet an insufficient rule-out test. IL-6 has shown a lower sensitivity and specificity in our study compared with previously reported results [9, 20].

Previous attempts at combining serum biomarkers using different calculation methods were published in the past. Qin et al. have shown that a combination of the D-dimer and the CRP/erythrocyte sedimentation rate (ESR) increases the sensitivity but leads to a significant decrease in specificity [19]. Bottner et al. reported high sensitivity and specificity for PJI detection both for CRP (95 and 96%) and IL-6 (95 and 87%) while their combination lead to a sensitivity of 100% and a specificity of 86% [21]. According to the conflicting results reported, no clear recommendation on the usefulness can be made [22]. A trend can be seen towards increased specificity with reduced sensitivity when comparing the mentioned results with our own multi-biomarker findings.

A multitude of synovial biomarkers with a good diagnostic performance has been described in previous years such as the leukocyte count; the percentage of polymorphonucleocytes; and the levels of CRP, α-defensin, calprotectin, leukocyte esterase, and IL-6 [23–26]. Large prospective series with a direct comparison of the diagnostic properties of serum and synovial biomarkers will be necessary in the future, especially to find optimal marker combinations for diagnostic algorithms.

A limitation to this study is the complex calculations necessary to combine the properties of multiple biomarkers. A possible solution would be an app-based calculation software for daily clinical practice. To minimize the interference bias, patients with known health conditions associated with elevated inflammatory markers were excluded. A drawback of this approach is the reduced generalizability of the results and the fact that the results could still be influenced by unknown secondary diseases. Another limitation is the heterogeneity of the study population with different PJI locations, extent of soft tissue affection, and consecutively the different surgical procedures performed.

## Conclusion

In this study, multiple biomarkers were tested for their diagnostic performance, with CRP and fibrinogen showing the best results regarding the AUC, accuracy, sensitivity, and specificity. It was not possible to further increase the diagnostic accuracy by combining multiple biomarkers using sophisticated statistical methods.

## Data Availability

Data and study materials are available on reasonable request.
